# Temporal representation of care trajectories of cancer patients using data from a regional information system: an application in breast cancer

**DOI:** 10.1186/1472-6947-14-24

**Published:** 2014-04-02

**Authors:** Gautier Defossez, Alexandre Rollet, Olivier Dameron, Pierre Ingrand

**Affiliations:** 1 Unité d’épidémiologie, biostatistique et registre général des cancers de Poitou-Charentes, Faculté de médecine, Centre Hospitalier Universitaire de Poitiers, Université de Poitiers, 6, rue de la milétrie, Poitiers, Cedex BP 199 86034, France; 2 Université de Rennes 1, IRISA UMR6074, Rennes, France; 3 INSERM, Poitiers CIC 802, France

**Keywords:** Epidemiology, Evaluation, Care trajectory, Temporal reasoning, Data integration, Cancer

## Abstract

**Background:**

Ensuring that all cancer patients have access to the appropriate treatment within an appropriate time is a strategic priority in many countries. There is in particular a need to describe and analyse cancer care trajectories and to produce waiting time indicators. We developed an algorithm for extracting temporally represented care trajectories from coded information collected routinely by the general cancer Registry in Poitou-Charentes region, France. The present work aimed to assess the performance of this algorithm on real-life patient data in the setting of non-metastatic breast cancer, using measures of similarity.

**Methods:**

Care trajectories were modeled as ordered dated events aggregated into states, the granularity of which was defined from standard care guidelines. The algorithm generates each state from the aggregation over a period of tracer events characterised on the basis of diagnoses and medical procedures. The sequences are presented in simple form showing presence and order of the states, and in an extended form that integrates the duration of the states. The similarity of the sequences, which are represented in the form of chains of characters, was calculated using a generalised Levenshtein distance.

**Results:**

The evaluation was performed on a sample of 159 female patients whose itineraries were also calculated manually from medical records using the same aggregation rules and dating system as the algorithm. Ninety-eight per cent of the trajectories were correctly reconstructed with respect to the ordering of states. When the duration of states was taken into account, 94% of the trajectories matched reality within three days. Dissimilarities between sequences were mainly due to the absence of certain pathology reports and to coding anomalies in hospitalisation data.

**Conclusions:**

These results show the ability of an integrated regional information system to formalise care trajectories and automatically produce indicators for time-lapse to care instatement, of interest in the planning of care in cancer. The next step will consist in evaluating this approach and extending it to more complex trajectories (metastasis, relapse) and to other cancer localisations.

## Background

### Care trajectories and guidelines

The care provided for patients with cancer requires a multi-disciplinary approach. Throughout the course of their illness, patients undergo a series of diagnostic investigations and surgical and medical treatments which are all occasions for contact with the care system. They follow care trajectories that vary according to geographical, temporal, institutional, medical, economic or social factors. The trajectory can become more complex in case of relapse or intercurrent illnesses, or it can be simpler if the patient is cured or stabilises. In addition, an optimal trajectory will depend on patient compliance, avoidance of redundancy in investigations, prevention of complications and delivery of appropriate therapies. Clinical practice guidelines have been developed for this purpose by groups of experts using updated information from medical research along the lines of Evidence-Based Medicine. The purpose of these guidelines is to reduce variability in practices, control costs, and above all improve the quality of care. Nevertheless, in actual operation, these recommendations are not always easy to implement in settings where multidisciplinary care involves several different professionals, or even several institutions. Indeed, the fact that recommendations exist does not always mean that they are put into practice, and there is the question of awareness of updates, and of the need to comply with them [1].

### Interest of modeling care trajectories

There is a need to describe, analyse and understand care trajectories by modeling the itineraries followed by patients. This process could also enable the evaluation of the appropriateness of care provision in relation to reference standards, and thus contribute to improving the organization of healthcare and to determining strategic choices [2]. The orientation of a patient through an optimized trajectory does indeed depend on satisfactory coordination among the different protagonists, and adequate planning of the care itself. In France, cancer care is evolving towards the formation of regional networks, so as to coordinate expertise, services and resource allocation. The regional health agencies (in France ARS –*Agences Régionales de Santé*) define and implement regional plans for hospital care aiming to meet requirements of accessibility and quality. Time is often a central element in care provision, which is why the reduction of the time-lapse to instatement of care is a major strategic orientation [3]. Modelling care trajectories offers an explicit process-oriented view of healthcare and will enable routine evaluation of the compliance of observed care trajectories with those set out in guidelines. The production of care trajectories and waiting-time indicators at the regional population level should contribute to improve care planning, ultimately ensuring that all patients have access to the appropriate treatment within an appropriate time-lapse.

### International context, computerization of medical data and interoperability

The observation of the actual functioning of the care system requires the existence of information systems suited to following up patient care trajectories in a given environment. Although numerous health information systems have been set up in developed countries, they were not specifically designed for this particular purpose. Every year the care system generates enormous amounts of data. The information required is fragmented and spread across a number of sources. Yet there are few tools able to mobilise and integrate this data for the purpose of describing and modeling a set of care trajectories that are characteristic of real-life patient trajectories.

The first work conducted in this area of health and on the scale of a population was certainly that concerning the classifications of patients, one of the first of which was the DRG (Diagnosis Related Groups). These were developed from the 1960s by Fetter [4], and the aim was to define comparable care provision groups in which individuals were expected to use the same level of hospital resources. These DRG led to numerous adaptations across the world, for instance the PMSI in France (*Programme de Médicalisation des Systèmes d’Information*), and to a whole body of related research [5-7].

The representation of care trajectories using data mining methods is a dynamic research area. These methods are useful to seek sequential patterns corresponding to the most frequent patient trajectories and to conduct formal analysis of concepts enabling the description of patient flows generating easily understandable graphical representations [6-15]. While methods of data mining aim to discover details in clinical trajectories or clinical pathways, the number of patterns discovered need to be restricted to the main stages defined by guidelines when the objective is to provide routine evaluation indicators of the compliance of observed care trajectories to guidelines. Clinical trajectories usually yield models restricted to a single piece of hospital information system. To our knowledge, there is no approach to date that has integrated multiple-source data from all hospitals and health structures involved in cancer care.

### Cancer registry data

Internationally, cancer registries have achieved a high degree of standardization of definitions, classification systems, methods of analysis of data. This has been important in ensuring the comparability of incidence data from cancer registries, and has enabled their increasing use in epidemiological, clinical and health services management studies [16]. Most modern cancer registries use information sources on computer media at some point in the data collection process. Work on the development of a multi-source information system centred on the patient has been conducted to collect relevant information for cancer case registration according to international rules [17]. The General Cancer Registry of Poitou-Charentes covers an administrative region of 1.8 million people in south-western France. The increasing availability of computerized information on cancer patients from pathology databases, hospital administration systems and other computerized data sources has led this registry to extend its computer systems to exploit these new opportunities.

### Objectives

The main objective of this study was to develop a representation of care trajectories over time for new cancer patients, using the data from the Poitou-Charentes Regional Cancer Registry information system, and to assess the reliability and validity of this representation by confronting trajectories derived from cancer registry data used by the algorithm with observed care trajectories documented from medical records. Since breast cancer is the most frequent cancer in women in France [18] and since it has been subject for many years to recommendations based on updated survey data [19,20], we chose to make a first illustration of an application of the algorithm in the setting of non-metastatic breast cancer.

### International registration rules

In compliance with national and international recommendations [17], since January 1st 2008 the General Cancer Registry of the Poitou-Charentes region (south-western France) has included all the incident cases of malignant tumour (haematological malignancies and solid tumours not including baso-cellular skin carcinomas), involving subjects regularly residing in the Poitou-Charentes region at the time of diagnosis.

### Information system

The information system uses an Oracle 11 g database. The data management procedures were developed in the SAS environment (version 9.3).

The database of the cancer registry is a time-stamped relational database storing medical data with the date of occurrence of every event (such as date of admission, date when a biopsy was performed). Temporal intervals (such as the start, end and duration of a treatment) are not directly available but can be computed from the data.

The cancer registration database results from the investigation of five types of electronic data sources in the Poitou-Charentes area and in neighboring départements (Data sources), where each data source comprises all facilities, centers or establishments involved in cancer care to ensure the completeness and the coverage of all areas of cancer care for patients residing in Poitou-Charentes area.

A single program manages data capture in the system, with distinction according to the formats of each source. The integration process of data from different sources for a given individual enables each source record to be linked to the individual to which it belongs, performed by an identity server (Computerised record linkage).

A single dataset has been created for the study of temporal representation of care trajectories by extracting from two of the five data sources (anatomical pathology data and hospital discharge data), which concern 93 facilities or hospitals.

### Data sources

The Registry has established collaboration with more than 100 partners in the Region and in surrounding departments. Five types of data sources, using various terminologies to describe diagnosis, are routinely collected:

• Anatomical-pathology (AP) data (pathology data) which includes free-text reports related to one or several ADICAP diagnostic codes (*Association pour le Développement de l’Informatique en Cytologie et en Anatomie Pathologique* – the French classification of lesions with topographical and histological axis [21]) (n = 28 facilities).

• Hospital discharge (HD) data recorded in the French medical information program (PMSI) [22] which includes ICD-10 diagnostic codes and CCAM medical procedure coded fields (*Classification Commune des Actes Médicaux* – the health insurance classification [23]) (n = 65 hospitals).

• Full reimbursement for cancer care granted by the French healthcare insurance service (IS) which includes ICD-10 diagnostic codes (n = 3 facilities).

• Data from cancer surveillance (CS) in cancer care centres (“*Centres de Lutte contre le Cancer*”) which includes ICD-O3 tumour codes. Cancer surveillance is a system of data collection in oncology promoted in France in 1975 by the National Federation of Cancer Care Centers. It enables the identification of topographic and histological diagnosis of tumours, their initial extension, as well as therapeutic and outcome data for all cases in a given cancer care center (n = 3 centres).

• Data from Multidisciplinary Consulting Meetings (MCM) which may or may not include ICD-10 diagnostic codes. Multidisciplinary Consulting Meetings are held and serve for exchange among specialists from different disciplines concerning the diagnostic and therapeutic strategies to adopt for cancer patients. They are an essential part of the organisation of cancer care (n = 2 regional networks). These meetings are known as Institutional Tumor Boards in the United States.

Each of these bodies regularly transfers the information required by the Registry in standardized structured encrypted files. A single program manages data capture in the system, distinguishing sources. The source data is the most detailed level of information available in the system.

### Computerised record linkage

A primary function in the operation of a cancer registry is to bring together information describing the same individual from a variety of data sources. Because multiple notifications of the same tumour are expected if several sources of information are used, effective procedures for linking data on the same individual are very important, minimizing duplicate registrations of the same tumour and/or individual. So data extractions include patient identity (name, surname, birthdate …). When loading data in the registry information system, patient identities are integrated into an identity server which by way of a semi-automated process identifies data related to one and the same patient. The patient identification process, based on computerised record linkage [16], enables automated linkage (using deterministic rules), detection of ambiguities, duplicate searches and manual patient grouping or separation. This process enables all eligible records from data sources integrated into the information system to be related to single patients.

### Cancer registration

A notification algorithm [24] determines, for each patient, the tumours that should be notified to cancer registry staff according to registration rules for multiple primary cancers [25] and already validated tumours. Then each case is manually checked by registry staff by visual inspection of information sources, assessing the need to refer to a patient’s medical record to register the tumour. Finally, all registered tumours contain relevant information, including date of diagnosis, ICD-O3 topography, ICD-O3 morphology and basis of diagnosis, and they are systematically related to the data sources. Following this step, the source data related to non-metastatic breast cancers can be selected on the basis of this relationship to enable the representation of the care trajectories.

## Methods

### Definitions

#### Care trajectory

The care trajectory is the term used to refer to the itinerary of a patient through the healthcare system and among the different actors over a continuous period from the onset of the illness to its resolution [26]. In cancer care, the care trajectory can comprise several successive episodes of care, and this entails a distinction between the phases of diagnosis, treatment, consolidation, cure or local or distant relapse.

#### Event and state

The representation algorithm developed is able to take account of each stage in the initial care provision for the tumour. The method chosen consists in modelling the care trajectory as an ordered succession of dated events aggregated into states, the granularity of which is defined from care provision guidelines.

An event is a phenomenon considered to be local and instantaneous, occurring at a particular time-point. An event is a time-stamped attribute (like a chemotherapy administration, regardless of events occurring before of after).

When the event lasts over time, we refer to a state, which is as a temporal interval with a start and an end. A state corresponds to the aggregation of repeated occurrence of several events over time according to their type and their chronology. A state corresponds to a phase or a period in treatment, defined as a stage in the initial care provision previously derived from guidelines.

For instance, the repeated occurrence of several events in chemotherapy before surgery treatment is equivalent to the definition of the state “neoadjuvant chemotherapy”.

#### Time scale

The time scale is determined by the information systems searched, which here, as in most areas of health, date the events occurring in the course of patient care according to calendar days. Consequently, the smallest measurable interval in the present study is the calendar day, and a point event is represented as an interval of one day.

### Identification of standard sequences of initial care provision for non-metastatic breast cancer

The main stages in the initial care provision for non-metastatic breast cancer identified in the national guidelines [20] are listed below:

1. A diagnostic period involving pathology investigations to confirm any suspected malignancy following clinical and/or radiological examination.

2. A period of pre-surgery treatment for infiltrating, voluminous and/or inflammatory cancers, indicated to obtain initial reduction of tumour volume. It may be envisaged for cancers that are non-operable at the outset, or according to the size of the tumour, so as to enable partial surgery . The reference treatments are hormone therapy and chemotherapy.

3. A surgery period, since as in the case of most cancers the treatment of breast cancer is ideally based on surgical removal of the tumour. This removal should be accompanied by homolateral axillary lymph node dissection. In case of an infiltrating tumour of small size and in the absence of palpable axillary adenopathy or suspect ultrasound scan image, the sentinel lymph node technique can be used. The surgical period also involves pathology examination of the surgical piece, conducted extemporaneously or following surgery, and this enables confirmation of malignancy.

4. A post-surgery medical period covering different therapies:

5. A radiotherapy period, involving irradiation of the tumour site and of axillary lymph nodes for non-metastatic breast cancer. The reference for all volumes treated is 50 gy in 25 fractions over a period of 33 days. The duration of radiotherapy can be increased by one or two weeks in cases where the patient presents a risk of relapse. It is therefore recommended that radiotherapy should be initiated not more than 12 weeks after surgery if no chemotherapy is planned. If chemotherapy is indicated, radiotherapy should be started not more than 5 weeks after the chemotherapy, and not more than 6 months after surgery. Neither immediate breast reconstruction nor prescription of targeted therapy should alter these time lapses.

– Chemotherapy, mainly anthracyclines and taxanes, is initiated 3 to 6 weeks after surgery, generally in 4 to 6 administrations 21 days apart, although patterns can vary according to treatment protocols. Adjuvant chemotherapy therefore lasts 9 to 15 weeks.

– HER2-targeted therapies, such as trastuzamab, are indicated when there is significant HER2 over-expression by the tumour. Depending on protocols, the administration cycle can vary, and can be either sequential, i.e. initiated after the chemotherapy, or concomitant with the administration of taxanes. The duration of administration is generally 1 year.

– Hormone therapy is only indicated in hormone-sensitive tumours. The duration of treatment is generally 5 years. Hormone therapy is initiated after any chemotherapy and radiotherapy.

**Figure 1:**
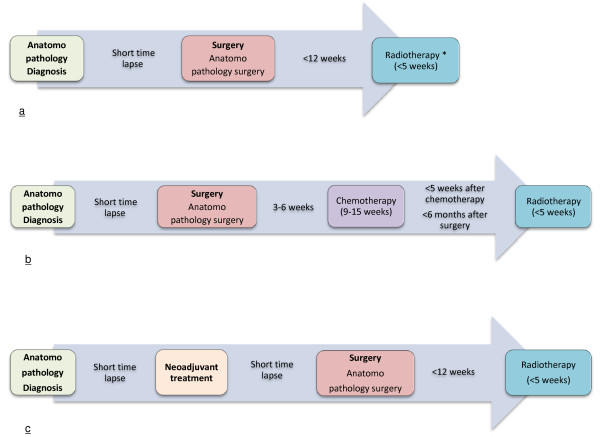
**Standard care sequences for non-metastatic breast cancer. a**: Care provision for non-metastatic breast cancer in case of good prognosis (* excepting patients undergoing total mastectomy and with no relapse factors). **b**: Care provision for non-metastatic breast cancer in case of presence of factors for poor prognosis. **c**: Care provision for non-metastatic breast cancer in case of voluminous, infiltrating and/or inflammatory cancer.

If these recommendations are synthesised, omitting targeted therapies and hormone therapy which are administered over long periods, it is possible to distinguish three standard care sequences for non-metastatic breast cancer, taking account of presence or absence of neo-adjuvant or adjuvant medical treatment (Figure 1). These standard sequences were constructed manually to define the different states in non-metastatic breast cancer management that are expected to occur in cancer care trajectories, and which are to be identified by the algorithm.

### Representation of care trajectories

The procedures for the temporal representation of care trajectories involve the following three stages:

1. Identification of tracer events in the care trajectory for each individual within the source datasets.

2. Chronological aggregation of events into states according to the level of granularity defined in step 1.

3. Representation of care sequences in a simple form (showing presence and order of states) and an extended form (including the duration of states) from which time lapses are calculated.

### Identification of tracer events

Events in the course of chemotherapy or radiotherapy are identified in HD data using ICD-10 diagnostic codes for chemotherapy (Z511) and radiotherapy (Z510) linked to the ICD-10 diagnostic code for breast cancer. For instance (Z511-C509) in source data refers to a “chemotherapy” event.

Surgical events – tumour removal, tumour and lymph node removal, and lymph node removal alone are identified in HD data from the 3rd alphanumerical character in CCAM codes (defining the action performed) and linked to ICD-10 code for breast cancer. For instance (C502-QEFA001) in the source data refers to the event “tumour and lymph node removal”.

Biopsy and surgery events are identified in AP data from the first character in ADICAP codes (identifying the sampling mode) for breast cancer. For example, (ADICAP-OHGSA7B2) in the source data refers to the event “surgery”.

In summary, HD data provide information on the nature of the surgical act performed, and AP data enables precise description of the act.

### Chronological aggregation of events into states

So as to be able to model sequences and position them on a temporal axis, we used a set of predefined relationships according to a linear approach which includes the notion of the time lapse [27,28]. The choice then was to retain only one characterised event per point in time before aggregating, rather than generating more numerous different states. This choice obeys the following hierarchy: tumour removal and/or lymph node removal > chemotherapy and/or radiotherapy > pathology sampling.

The aggregation takes account of the chronology of events and complies with the following two rules:

– If an event occurs between two records that would normally have been aggregated, the aggregation is not performed. For instance, if the patient undergoes surgery between two chemotherapy sessions, the aggregation of these two sessions into a “chemotherapy” state does not occur.

– If the time-lapse between two events of the same type is too long, the aggregation is not performed, and this enables the differentiation of two distinct care episodes (six months between two chemotherapy administrations, one month between two radiotherapy sessions, three months between two surgical acts, three months between two pathology samples). For instance if the patient has 3 months’ chemotherapy following surgery, and then 1 year later another 3 months chemotherapy for a relapse, the algorithm will differentiate the two successive chemotherapy periods, and they will not be aggregated.

The rules for aggregating events into states are presented in Table 1. Because the main stages in the initial care provision for the tumour are defined from care provision guidelines, the relevance and completeness of the states illustrated in Table 1 are ensured by expert consensus.

**Table 1:** Main rules for aggregation of events into states

State code*	**State**	**Type of event to aggregate (time-lapse between two events for aggregation)**	**Dating**
A	AP	≥ 1 pathology investigations (less that 3 months apart)	Sampling Date
			If several:
			Start: date of first investigation
			End: date of last investigation
C	SURG	≥ 1 surgical acts (less than 3 months apart)	Date of the act
			If several:
			Start: date of first surgery
			End: date of last surgery
D	SURG_AP	≥ 1 surgical acts (less than 3 months) apart AND ≥ 1 pathology investigations	Date of the act, or else date of the sample.
			If several:
			Start: date of first surgery
			End: date of last surgery
N	CT_NEO	≥ 1 administrations of chemotherapy occurring before the first surgery	Start: date of the first administration
			End: date of the last administration
K	CT	≥ 1 administrations of chemotherapy (less than 6 months apart)	Start: date of the first administration
			End: date of the last administration
R	RT	≥ 1 radiotherapy sessions (less than 1 month apart)	Start: date of the first session
			End: date of the last session
O	CT_RT	Period of concomitant radiotherapy and chemotherapy	Start: date of the first session of intercurrent RT or CT
			End: date of the last session of intercurrent RT or CT

*Each state is recoded by a character so as to represent the overall sequence in the form of an ordered chain of characters.

It should be noted that a distinction is made for the concomitant administration of radiotherapy and chemotherapy in one and the same report. This situation can arise when HER2-targeted therapy is administered on the occasion of a visit by the patient for radiotherapy. The algorithm produces the specific state “chemotherapy-radiotherapy” (CT-RT).

### Representation of sequences

**Figure 2:**
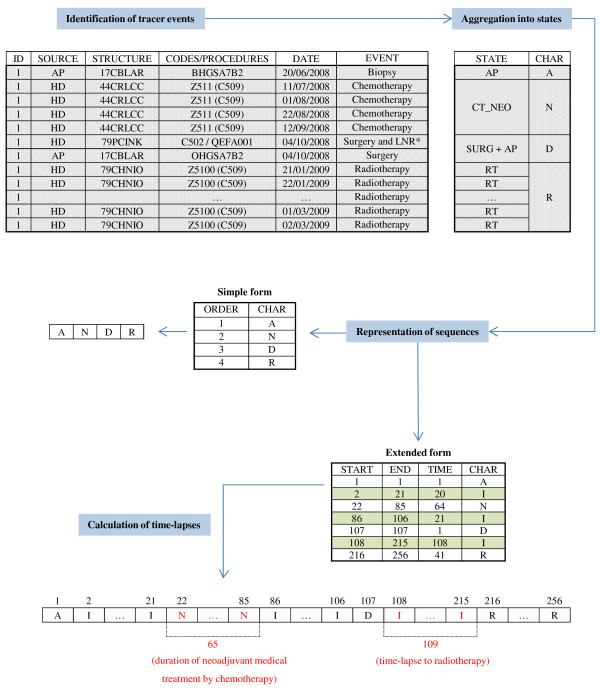
**Algorithm representing care trajectories.** Figure 2 presents an illustration of the representation of the care trajectory for a patient with infiltrating ductal adenocarcinoma of the breast. The diagnosis was established on examination of a biopsy by pathologists in a laboratory in La Rochelle, Charente-Maritime (17CBLAR). Treatment was a sequence of neo-adjuvant chemotherapy (4 sessions) in Nantes cancer center, Loire Atlantique (44CLRCC), followed by partial mastectomy and lymph node removal performed in Niort private hospital, Deux-Sèvres (79PCINK) and then 30 sessions of irradiation in Niort general hospital, Deux Sèvres (79CHNIO). * LNR: Lymph Node Removal, (I) The extended form integrates periods without any event noted I, where the start corresponds to the day following the previous state and the end to the day preceding the following state.

The sequences of states produced by the algorithm are stored vertically in SPELL format [29], where a line represents a state and each state is characterised by a start and an end. The data for each patient is then transposed so as to present the whole sequence in the form of an ordered chain of characters. An example of a representation of a care trajectory for a patient with infiltrating ductal adenocarcinoma of the breast is shown in Figure 2.

The sequences produced are available in two forms:

• A simple form in which the presence and order of the states are shown.

• An extended form which integrates the duration of states. This form includes periods without any event. The chains of characters are extended by repeating each character according to the duration of the state.

The choice to represent care trajectories as chains of characters where the length is directly proportional to the duration of care provision enables simple calculation of time-lapses via the use of PERL regular expressions [30,31].

### Evaluation of the representation of care trajectories

**Figure 3:**
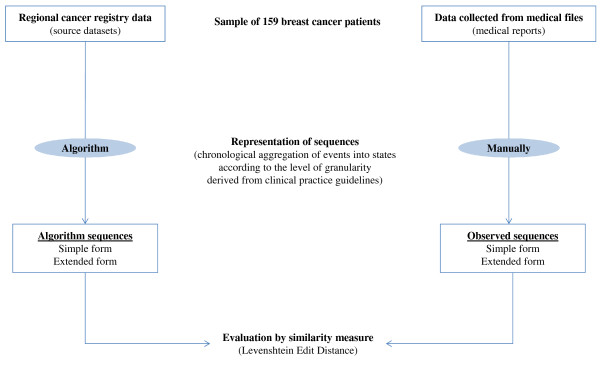
**Strategy for evaluation of temporal representation of care trajectories.** Target trajectories as observed sequences: trajectories manually documented from medical files. Algorithm trajectories as algorithm sequences: trajectories automatically produced by the algorithm from cancer registry source data.

The quality of the representations of care trajectories produced by the algorithm was assessed on a sample of patients for whom the care trajectories were constructed manually from data collected in the medical record (validation dataset). This dataset served as the reference standard for the evaluation of the representation of care trajectories. Each sequence was generated twice and independently from regional cancer registry data (automatically as “algorithm sequences”) and from medical record (manually as “observed sequences”). The performance of the algorithm was assessed by confrontation of the sequences generated by the algorithm with the observed sequences for the patients (Figure 3).

### Study population

The sample was formed by random selection stratified on TNM extension stage at diagnosis. A minimum of 50 patients per stratum (TNM stages I, II and III) was requested to conduct the evaluation of the algorithm in order to cover a variety of care trajectories of patients with breast cancer. The sample comprised 159 subjects with unilateral non-metastatic breast cancer (TNM stages I, II and III) diagnosed in 2008 among patients residing in the Poitou-Charentes region at the time of diagnosis, and who received all their care in three of the five health territories in the region covered by the public radiotherapy unit (Vienne, Deux-Sèvres, Charente-Maritime Sud).

### Reconstruction of observed sequences

Whereas the algorithm-based sequences were produced using solely the coded data from two electronic sources, the 159 observed sequences were manually reconstructed from the medical records, which collect all patient information produced and documented by staff involved in patient care. For this work the cancer registry staff systematically collected a copy of original pathology reports, surgery reports, hospitalisation reports or other relevant reports needed for the documentation of the main events in patient care trajectories. Each event occurring between diagnosis and the end of initial care provision for cancer was precisely documented (date and result of pathological investigation, date and nature of the surgical act, dates of start and end of chemotherapy, radiotherapy, HER2-targeted therapy, hormone therapy, number of sessions, alterations in treatment, occurrence of complications). This documentation may be derived from several different institutions depending on the number of establishments frequented by the patient.

The states making up the sequence were then manually captured in a single data table using the same rules implemented by the algorithm for the production of states. The observed trajectories were structured according to the simple form and the extended form, to enable confrontation with the trajectories produced by the algorithm.

An endpoint for the end of treatment for each patient was determined manually from the last state in the initial care provision for the tumour. The endpoint of a trajectory can correspond to the death of the patient if it occurs before the end of the initial course of treatment of the cancer.

### Similarity measures

A similarity measure was performed on all pairs of trajectories using the Levenshtein Edit Distance (LED) [32], which enables comparison of chains of characters of different lengths using identical cost operations. The LED is equal to the minimum number of characters that need to be added, removed or replaced to switch from one chain of characters to the other. Each of these elementary operations is associated with a cost equal to 1. The LED function is available on SAS software using the function COMPLEV.

### Authorization and accreditation of the general cancer registry of Poitou-Charentes

In conformity with French law, the collection and analysis of medical data by the General Cancer Registry of Poitou-Charentes has received the approval of the French regulatory authorities: the *Comité Consultatif sur le Traitement de l’Information en matière de Recherche dans le Domaine de la Santé* and the *Commission Nationale Informatique et Libertés* (authorization number 907303).

In France, the *Comité National des Registres* (CNR) evaluation grids to be applied for the accreditation of registries include not only the methods used and the quality of the records, but also the use made of the data, and the interest and the originality of the research work conducted [33]. The General Cancer Registry of Poitou-Charentes has been approved by the French CNR since January 2013 based on the 2008 and 2009 registered data.

## Results

### Study population

The sample comprised 159 subjects with non-metastatic breast cancer. The mean age at diagnosis was 64.0 ± 15.3 (range 30–94). Fifty-two per cent were between the ages of 50 and 75, corresponding to the age group eligible for breast cancer screening from 2008. Fifty-five patients were TNM stage I, 52 were TNM stage II and 52 were TNM stage III.

### Description of the sequences observed in the sample

The sequences observed in the sample are shown in Table 2. An individual care sequence belongs to one of the three categories illustrated in Figure 1 when the main tracer events are present in the same order as in the standard care sequence. Variants can be expected according to the presence or the absence of a pathology investigation, such as biopsy (“A”) or a HER-targeted therapy (“O”). Otherwise, sequences are classified as non-standard sequences, and result in the particular cases being described as the fourth category “Other (non-standard sequences)”.

**Table 2:** Description of observed (actual) sequences in the sample

**Care sequences**	**Numbers**	**Percentage**
*Care provision for non-metastatic breast cancer in case of good prognosis (Figure *1* a)*	*81*	*51%*
ADR	47	30%
AD	16	10%
DR	13	8%
D	5	3%
*Care provision for non-metastatic breast cancer in case of presence of factors for poor prognosis (Figure *1* b)*	*54*	*34%*
ADKR	42	26%
ADKOK	8	6%
DKR	2	1%
DKOK	2	1%
*Care provision for non-metastatic breast cancer in case of voluminous, infiltrating and/or inflammatory cancer (Figure *1* c)*	*8*	*5%*
ANDR	8	5.0
*Other (non standard sequences)*	*16*	*10%*
A	6	5%
ADK	2	0-1%
AK	2	0-1%
AR	2	0-1%
ADKDR	1	0-1%
ANDRK	1	0-1%
DK	1	0-1%
DKDKOK	1	0-1%

A Pathology investigation.

D Surgery and pathology examination of the surgical pieces.

N Neo-adjuvant chemotherapy.

K Chemotherapy.

R Radiotherapy.

O Concomitant chemotherapy and radiotherapy.

Ninety per cent of these sequences were identifiable in the three standard sequences derived from recommendations (Figure 1). All the sequences involving a period of concomitant chemotherapy and radiotherapy (state “CT-RT” coded O) correspond to patients treated with HER2-targeted therapy.

More than half the observed (actual) sequences (51% or 81 patients) corresponded to the standard “ADR” sequence which combines pathology examination for diagnostic purposes, a surgical act for removal of tumour and/or lymph nodes, together with pathology examination of the surgical pieces, and radiotherapy treatment. Among these 81 sequences, 21 patients had total mastectomy, with biopsy (“AD”) or without (“D”), and no relapse factor and hence absence of radiotherapy, while 13 patients had partial or total surgery on the basis of clinical parameters, and relapse risk factors providing indication for radiotherapy (“DR”).

One third of the observed sequences (34% or 54 patients) corresponded to the standard “ADKR” sequence, combining diagnostic pathology investigation, a surgical act for removal of tumour and/or lymph nodes alongside pathology examination of the surgical pieces, chemotherapy treatment and radiotherapy treatment. Two patients underwent surgery at the outset (“DKR”) and 10 were treated with HER2-targeted therapy, with biopsy (“ADKOK”) or without (“DKOK”).

Eight patients followed the “ANDR” standard sequence (5%) which associates diagnostic pathology examination, neo-adjuvant chemotherapy, a surgical act for removal of tumour and/or lymph nodes, with pathology investigation of the surgical pieces, chemotherapy and then radiotherapy. Among these 8, 7 had a large and/or inflammatory carcinoma, and one patient expressed the wish to preserve the breast.

Ten per cent of the remaining sequences (16 patients) were particular cases:

– Ten patients who refused surgery, or whose advanced age constituted a counter-indication for surgery (“A”, “AK”, “AR”).

– For three patients, with and without secondary total mastectomy (small breasts and areas surrounding the exeresis not clear) the decision, justified in MCM, was to administer chemotherapy but not radiotherapy (“ADK”, “DK”).

– Two patients having undergone secondary surgery at a late stage (“DKDKOK”, “ADKDR”).

– One patient who received HER2-targeted chemotherapy at a late stage “ANDRK”).

### Performance of the algorithm

#### Pair similarity in simple sequences (simple form)

Ninety-eight per cent of the sequences generated by the algorithm were similar to those observed when generated manually in terms of presence and ordering of states, i.e. there were only three dissimilar sequences among the 159 (LED = 1). These dissimilarities were related to absence of biopsy pathology report for one sequence (algorithm sequence “K” versus observed sequence “AK”), and absence of pathology report for the surgical pieces for two sequences (algorithm sequence “AC” versus observed sequence “AD”).

#### Pair similarity in extended sequences (extended form)

Eighty-eight per cent of the identical sequences with respect to presence and ordering of states were also similar to one day for the duration of the states, giving 18 dissimilar sequences out of 159 (LED median = 7.5, range 1–51). The dissimilarities between sequences were in 89% (16 of 18 patients) date errors, or absence of coding for chemotherapy sessions (LED 21–51) or radiotherapy sessions (LED 1–3) at the start or the end of a state. One patient had no HD data on the lymph node removal she underwent secondarily (LED = 8). Finally, one patient presented a date error of one day in the date of surgery (LED = 1).

The percentage of match of pairs of extended sequences was 94% (10 dissimilar sequences out of 156) to the nearest 3 days (LED = 3).

## Discussion

This study presents a method for the temporal representation of care trajectories for patients with non-metastatic breast cancer using data from a regional multi-source information system. The method proposed enables identification of the main tracer events in a care trajectory, and also integrates the duration of each event, and the time-lapse between events making up the trajectory. The results of the experiment show that the algorithm is able to reconstruct the care trajectories automatically from the registry data without implementing a collection of relevant information in medical records, which would be very resource-consuming in this setting.

### Performance of the algorithm in the ordering of states

The ordering of treatment periods within the care sequences was correctly represented in 98% of cases. The three discrepancies observed were linked to absence of pathology evidence in the Registry data source, corresponding to non-coded sampling procedures in the pathology data (the practitioner did not code the tumour), or to coding errors or inconsistencies. Two of the dissimilar sequences nevertheless comprised all the treatment periods, because the absence of a pathology report on the surgical piece led to the creation of an intermediate surgery state in the sequence (“C” - surgery alone - rather than “D” - surgery and pathology evidence). An earlier study [34] implemented a text categorisation method using a machine-learning technique for the purpose of automatically categorising pathology reports solely on their content, which has demonstrated very good performances. It is therefore likely that the performance of the algorithm could be improved further by adding a supplementary check of the coding of pathology reports.

### Performance of the algorithm on the duration of states

The performance of the algorithm relating to the duration of states was 88% to the nearest day, and 94% to three days, suggesting that the time-lapse indicators are excellent for the main tracer events in a care trajectory. The choice of representing care trajectories as chains of characters where the length is directly proportional to the duration of care provision makes it possible to extract and accurately calculate time-lapses in care trajectories as required, on the basis of regular expressions. Each regular expression needs to be drafted and adapted to the type of time-lapse or treatment duration to be calculated, thus enabling the main time-lapses to be produced for use in planning care in cancer units.

The dissimilarities between sequences were in 90% of cases related to dating errors or absence of coding for chemotherapy or radiotherapy sequences occurring at the start or at the end of a state. The variability of the dissimilarities observed was mechanically related to treatment patterns. The dissimilarities ranged from 21 to 51 days according to the sequence when the coding errors were linked to chemotherapy sessions, since the reference administration pattern is 9 to 15 weeks. Dissimilarities were thus much smaller when the coding errors concerned radiotherapy sessions (1 to 3 days depending on sequences) since the reference pattern for the volumes treated is 50 Gy in 25 fractions over 33 days. Conversely, coding errors that persisted in the middle of a state before aggregation had no impact on its duration.

### Limitations

First, one limitation of this study is its restriction to only three of the 5 health “territories” in the Poitou-Charentes region, because of absence of data available relating to radiology in private practice. Indeed, private radiology units bill their activities on the basis of CCAM-coded acts performed, and they are not covered by PMSI. The integration of this activity into a database is essential because the share of radiotherapy sessions performed on a profit-making basis by private facilities was estimated to be 55% over the territory as a whole in 2002 [5]. There are no consequences on the performances of the algorithm because the evaluation was conducted on a sample of patients who received all care in three of the five health territories in the region covered by the public radiotherapy unit. However, absence of radiotherapy data from private establishments in the cancer registry does not enable an evaluation of initial care trajectories extended to all breast cancer patients living in Poitou-Charentes area to be produced. We can however hope that this data will be integrated into the Registry database in the near future, since a reorganisation of the funding system for private-practice radiology is underway, and the units that at present code their activities according to CCAM will soon be required to produce standardised discharge reports as under PMSI [35].

Second, representing care trajectories by way of a linear approach generates difficulties when therapies are concomitant and when they are administered over long periods. These problems can be managed by creating composite states, with the risk of increasing the number of different sequences. In the ICD-10 there is no distinction between classic chemotherapy and HER2-targeted therapy, since both are coded Z511 for anti-tumour chemotherapy. A supplementary state “CT-RT” was therefore created to enable the representation, in this particular instance, of a treatment period relating the HER2-targeted therapy and concomitant radiology. This choice proved to be opportune, since all the sequences associating HER2-targeted therapy and radiotherapy were correctly identified and restored by the algorithm. Obviously, the creation of composite states will be discussed for each application of the algorithm to new cancer localization.

Third, not all the relevant sources of cancer data were included in modelling the trajectories. As can be seen from the 10% non-identifiable sequences among standard sequences from the different guidelines, the MCM are useful for the identification of specific instances relating to hospital care protocols, decisions linked to comorbidity, or patient choices. The integration of the MCM into the representation of sequences is also useful for the production of time-lapses to post-surgery therapeutic proposals. This source of data, although available at the time of study, was not assessed for the exhaustiveness of its integration into the Registry information system and therefore has not been included in the analysis.

Fourth, today the Registry does not routinely collect data relating to mammography, but it is working along these lines with screening centres in the Poitou-Charentes region. Bilateral mammography, the reference investigation in case of clinical warning signs or in screening campaigns, would be a very valuable element to include at the start of a trajectory, so as to analyse the time-lapse to diagnosis (time between mammography and biopsy) or the time-lapses more generally over the whole duration of care provision (for instance the time-lapse between mammography and the end of radiotherapy).

### Comparison with the literature

Our modelling approach drew from work conducted in the socio-economic sphere using sequence analysis methods known as optimal matching, also known as sequence alignment, originally developed for rapid analysis of proteins and DNA sequences [36]. The first optimal matching algorithms appeared at the start of the 1970s, and their first application in the social sciences dates back to the article by Abbott and Forrest and their application to historical data [37,38]. Many techniques and tools, such as data mining, workflow mining or process mining [6-15] give a conceptual framework for clinical trajectory analysis. The aim of clinical trajectories is to offer a flowchart format for the decisions to be made and the care to be provided for a given patient or patient group. All published methods sought to enumerate regular medical behaviors that are expected to occur in patient-care trajectories from clinical workflow logs recorded by hospital information systems. But none to our knowledge has performed an application on patient care data in order to give an assessment of which regular medical behaviors occur in patient-care trajectories in real-life, rather than those expected. Moreover, they frequently implement models that provide too much detail to give a concise and comprehensive summary of the trajectory and are usually restricted to a single hospital information system. Our work focused on the representation of care trajectories through the main stages in the initial care provision for non-metastatic breast cancer identified in current updated guidelines, based on relevant and accurate information from medical source records from all hospitals and health facilities involved in cancer care on a large geographical scale. To our knowledge this work is the first to assess the reliability and the validity of an algorithm for the representation care trajectories from cancer registry data. The method proposed in this work responds to needs expressed by institutions and health professionals of routine indicators aimed to improve the quality of patient care.

The structured organisation in the form of a relational time-stamped database in the Poitou-Charentes cancer Registry was well-suited to the application of these techniques. The value of our study is that it models the care trajectory from on-going routine collection of data for exhaustive recording of incident cases of cancer. This approach is useful for routine manual registration of incident tumours, because it makes relevant information available to registry staff, information that is dated and presented in chronological order, facilitating visual inspection of the case and the identification of structures possessing complementary information, thus enabling cases to be recorded according to international standards.

The routine production of care trajectories in cancer and waiting-time indicators for the purpose of evaluation, which are by-products of tumour notification to the Registry, opens up new perspectives in terms of cover, scope for comparison in time and space, and costs of producing information. A recent French study presented an overview on regional level of time-lapses between the different acts and key steps in the trajectories of patients with breast and lung cancer [39]. This study underlined the time required to collect data, imposed by the scatter of information for a given care trajectory, and the difficulties linked to the heterogeneity of data collection methods and practices, so that routine short-term follow-up of observed waiting times is not possible. Similar approaches have been implemented in other countries, for example in Ontario province, Canada, in the UK, or in New Zealand [40-43]. These countries have undertaken to study waiting times and estimate reductions in time-lapses to treatment, which has become one of the aims in their cancer Plans.

Certain specialised registries systematically record data concerning the initial treatment. This is the case, for instance, for breast cancer in the breast and gynaecological cancer Registry of the Côte d’Or area in France. This Registry used its database to supply information for a study conducted within the Francim network of cancer registries entitled “From diagnosis to first treatment: time-lapse to instatement of care for cancers recorded by the specialised registries in the Francim network 1999-2008”, recently published by Inca [44]. It showed that there were variations in time-lapse to treatment over this period.

However, the majority of the general registries do not systematically collect information on care provision, and do not therefore have any scope for generalising these indicators unless a specific survey is conducted [45].

### Perspectives

The routine production of these indicators will enable regular assessment of the match between care provided and official guidelines, the evaluation of time-lapse to treatment, and comparison of results with those reported in the international literature. This method can be applied by other cancer registries, conditionally on the availability of coded data sources using international classification of disease (ICD-O-3, ICD-10).

The results presented in this study are in favour of a continuation of this work on other cancer localizations. Before implementing the algorithm on the population-based cancer registry database for a new cancer localization, a further analysis of national guidelines and an evaluation on a new sample of patients are required. There are localisations where reducing time-lapse to treatment instatement is a major strategic orientation, and where providing care complying with guidelines requires the histological type to be identified, as in lung cancer. There are also rarer pathologies such as multiple myeloma, where the application of the algorithm would provide an overall picture of the organisation of the care trajectory, the diversity of trajectories according to patient characteristics and the different players in the care process. In our study the analysis was restricted to non-metastatic breast cancer and to the initial care provision for this cancer. Although rules were applied to avoid aggregating two events of the same type belonging to two different care episodes, the analysis needs to be extended to care episodes following the initial care trajectory, such as care relating to the occurrence of local or distant relapse, thus approaching the notions of cure, relapse, remission and consolidation.

Finally, one present limitation inherent in the representation and analysis of care trajectories in cancer is the absence of data on radiotherapy for private facilities, and the absence of data on the administration of treatment outside the hospital environment (oral chemotherapy and other). Subsequent work should therefore study the feasibility of obtaining and integrating SNIIR-AM data (French national insurance information system) to improve the representation of care trajectories among cancer patients.

## Conclusions

This study presents a temporal representation of care trajectories in the form of a sequence of states ordered in time. The system enables accurate, routine identification of key dates and events in care trajectories, despite the fact that the data is initially fragmented across numerous sources in different territories. This information is collated in a single base from which it can be readily extracted and exploited. The crossing of the trajectories produced with certain clinical data will enable routine evaluation of the compliance of observed care provision trajectories with those set out in guidelines. The production of indicators of this sort will contribute to significantly improving care planning at regional level, ultimately ensuring that all patients have access to the appropriate treatment within an appropriate time-lapse.

## Consent

Patients were individually informed before the start of data collection of the nature of the information provided, the purpose of data processing, and their right of access, rectification or objection in conformity with the French law. The collection and analysis of medical data by the General Cancer Registry of Poitou-Charentes has received the approval of the French regulatory authorities: the Comité Consultatif sur le Traitement de l’Information en matière de Recherche dans le Domaine de la Santé and the Commission Nationale Informatique et Libertés (authorization number 907303).

## Abbreviations

ADICAP: Association pour le Développement Informatique en Cytologie et Anatomie Pathologique; CCAM: Classification Commune des Actes Médicaux; CNR: Comité National des Registres; DRG: Diagnosis related groups; ICD-10: International classification of disease, 10th edition; ICD-O3: International classification of disease, oncology 3rd edition; MCM: Multidisciplinary consulting meeting; PMSI: Programme de Médicalisation des Systèmes d’Information.

## Competing interests

The authors declare that they have no competing interests.

## Authors’ contributions

GD and AR performed the data collection, data analysis and data interpretation. GD wrote the manuscript. AR, OD and PI contributed to data interpretation and drafting of the manuscript. All authors read and approved the final manuscript.

## Pre-publication history

The pre-publication history for this paper can be accessed here:

http://www.biomedcentral.com/1472-6947/14/24/prepub
